# Modifiable factors of depressive-symptom trajectories from caregiving through bereavement

**DOI:** 10.1186/s12904-022-01045-9

**Published:** 2022-09-07

**Authors:** Fur-Hsing Wen, Wen-Chi Chou, Po-Jung Su, Ming-Mo Hou, Wen-Chi Shen, Mei Huang Hsu, Siew Tzuh Tang

**Affiliations:** 1 Department of International Business, Soochow University, Taipei, Taiwan, ROC; 2 Division of Hematology-Oncology, Chang Gung Memorial Hospital at Linkou, Tao-Yuan, Taiwan, ROC; 3 College of Medicine, Chang Gung University, Tao-Yuan, Taiwan, ROC; 4 School of Nursing, Chang Gung University, 259 Wen-Hwa 1st Road, Kwei-Shan, Tao-Yuan, 333 Taiwan, ROC; 5 Department of Nursing, Chang Gung Memorial Hospital at Kaohsiung, Kaohsiung, Taiwan, ROC

**Keywords:** Depressive symptoms, Trajectories, Modifiable factors, Family caregivers, Bereavement, Caregiving, End-of-life care, Cancer

## Abstract

**Background/purpose:**

The purpose of this secondary-analysis study was to identify never-before-examined factors associated with distinct depressive-symptom trajectories among family caregivers from end-of-life caregiving through the first 2 bereavement years.

**Participants/methods:**

Participants (*N*=661) were family caregivers who provided end-of-life caregiving for terminally ill cancer patients. Multinomial logistic regressions were conducted to identify modifiable factors associated with caregivers^’^ seven previously identified depressive-symptom trajectories: minimal-impact resilience, recovery, preloss-depressive-only, delayed symptomatic, relief, prolonged symptomatic, and chronically persistent distressed. Drawing from the stress-appraisal-coping model, modifiable time-varying factors associated with distinct depressive-symptom trajectories were examined in three domains: (1) stressors, (2) stress appraisal, and (3) available resources (internal coping capacity and external social support)*.*

**Results:**

Profound objective caregiving demands were associated with caregivers’ increased likelihood of belonging to more distressing depressive-symptom trajectories than to the minimal-impact-resilience trajectory. But, stronger negative appraisal of end-of-life caregiving increased odds of caregiver membership in preloss-depressive-only and relief trajectories over the recovery, delayed, and prolonged-symptomatic trajectories. Stronger internal coping capacity and perceived social support buffered the tremendous stress of end-of-life caregiving and permanent loss of a relative, as evidenced by higher odds of being in the minimal-impact-resilience and recovery trajectories.

**Conclusion:**

Family caregivers’ distinct depressive-symptom trajectories were linked to their preloss caregiving demands, appraisal of negative caregiving impact, personal coping capacity, and perceived social support. Our results highlight actionable opportunities to improve end-of-life-care quality by boosting family caregivers’ coping capacity and enhancing their social support to help them adequately manage daily caregiving loads/burdens thus relieving the emotional toll before patient death and throughout bereavement.

**Supplementary Information:**

The online version contains supplementary material available at 10.1186/s12904-022-01045-9.

## Introduction

Family caregivers are a critical constituent in the end-of-life (EOL)-cancer-care system because they bear the brunt of caregiving for terminally ill cancer patients [1, 2]. In the absence of caregivers, society would pay substantial additional costs for healthcare services [1, 2]. These externalized costs come with great physical [3, 4], psychological [5], and financial [3, 4] consequences for caregivers. Though most caregivers adjust to the loss of their relative over time [6], patient death brings no redress for a minority of bereaved with profound, negative EOL-caregiving effects sustaining well into bereavement [7]. For instance, the bereaved face more enduring serious mental problems than the general population [8], like depressive disorders that strain one’s personal health [9], family/social networks [9], workplaces [9], and healthcare systems [9, 10].

Caregiving for and losing a relative is among the most devastating events one may experience [11], though caregiving experiences and grief reactions vary substantially across cultures [6]. For example, under the most fundamental moral Confucian duty, family caregivers in Asian cultures feel obligated by filial piety to assume caregiving roles to repay for the life and care they received [12], whereas with regard to cultural grief reactions, individuals from non-western cultures tend to somatize their psychological distress (e.g., depressive symptoms) [6]. Examination of caregiving experiences and grief reactions must account for cultural context.

Furthermore, caregivers respond heterogeneously to caregiving/loss events as shown by belonging to distinct depressive-symptom trajectories [11, 13, 14, 15, 16, 17, 18, 19, 20, 21, 22, 23, 24]. Most caregivers overcome these highly stressful changes, whereas a minority maladaptively adjust [11, 13, 14, 15, 16, 17, 18, 19, 20, 21, 22, 23, 24]. Based on the stress-appraisal-coping model [25] and systematic reviews [26], a potentially threatening caregiving or bereavement event is not necessarily stressful per se but is mediated by a complex web of (1) contextual factors (e.g., social-demographics), (2) stressors (e.g., caregiving demands), (3) stress appraisal (e.g., appraisal of caregiving impact), and (4) available internal/external resources (e.g., coping capacity/social support) which determine adjustment outcomes*.* Opportunities to improve EOL-care quality begin with identifying caregivers at risk of profound/prolonged/persistent depressive symptoms from EOL caregiving to bereavement*.*

Factors associated with distinct depressive-symptom trajectories have been examined independently during caregiving [13, 14, 15, 16] or bereavement [17, 18, 19, 20, 27], while focusing largely on demographics and social support. Caregiving and bereavement are ongoing, intertwined processes of caring for a family member, dealing with the relative’s death, and grieving for the deceased [28, 29]. Indeed, a “bereavement effect” begins long before death [30] but caregiving factors have only been examined either at baseline [18] or right before patient death [20], as potential predictors of depressive-symptom trajectories during bereavement.

The relationship between EOL caregiving and bereavement may be cumulative (wear-and-tear model) or abating (relief model) [28, 29]. The wear-and-tear model [28] suggests that exposure to the chronic stresses of EOL caregiving depletes caregivers’ emotional and social resources, exacerbating the impact of losing a relative, whereas the relief model indicates that after the difficulties of EOL caregiving, patient death brings the caregiver relief and thus better bereavement outcomes [29]. To disentangle the inconclusive role [6, 7] of these competing models, factors moderating bereavement adjustment must be examined before and after patient death. This study was undertaken to examine modifiable factors associated with our previously identified distinct depressive-symptom trajectories from EOL caregiving through the first 2 bereavement years among caregivers of terminally ill cancer patients to highlight actionable opportunities to improve EOL-care quality.

## Methods

### Research design and sample

Data for this secondary-analysis study were from two institutional review-board-approved (98-0476B, 101-0898A3) longitudinal studies on associations of EOL caregiving with and the effectiveness of an advanced care planning intervention on caregivers’ depressive symptoms before and after patient death [31]. Details have been reported on participant eligibility, subject enrollment and participation in postloss surveys (including validation of between-study and between-participation-status homogeneity), measures, and identification of distinct depressive-symptom trajectories from EOL caregiving through the first 2 bereavement years [31]. In brief, adult caregivers of terminally ill cancer patients (recognized as having a progressing, metastatic cancer by their oncologist) were recruited by convenience from a medical center in Taiwan from 2010 to 2017 and followed through their first 2 bereavement years (end July 2020). Detailed participant characteristics are Table 5 in Appendix 1. As reported in our published article [31], among those 661 caregivers who participated in bereavement surveys, numbers (bereavement follow-up rates) of bereaved caregivers who participated at 1, 3, 6, 13, 18, and 24 months postloss are as follows: 616 (93.2%), 520 (78.7%), 510 (77.2%), 471 (71.3%), 418 (63.2%), and 382 (57.8%), respectively. Among these 661 caregivers, 187 (28.3%), 227 (34.3%), 280 (42.4%), 361 (54.6%), 470 (71.1%), and 625 (94.6%) provided data during each of the last 6 months preloss, respectively.

### Data collection

Caregivers were interviewed in person for preloss caregiving experiences (i.e., depressive symptoms, stressors, stress appraisal, internal/external resources, and prognostic awareness) at enrollment and approximately monthly thereafter while providing EOL caregiving until they declined to participate. Time invariant covariates were assessed at baseline. Bereaved caregivers were surveyed for depressive symptoms at 1, 3, 6, 13, 18 and 24 months postloss, whereas internal/external resources were measured at 1-month postloss only.

### Measures

#### Outcome variable

Depressive symptoms were measured with the 20-item Center for Epidemiologic Studies Depression Scale (CES-D) [32], with four subscales: positive emotions, depressive emotions, physical activities, and social difficulties. Items are scored for frequency during the past week by a 4-point Likert scale from 0 to 3. Total scores range from 0 to 60; higher scores indicate more depressive symptoms [32]. CES-D scores >16 are considered the threshold for severe depressive symptoms [32]. Prevalence of severe depressive symptoms among the 661 participants increased substantially over the patient’s last 6 months as the patient’s death approached from 9.1% to 69.0% and decreased steadily over the first 2 bereavement years from 69.1% at 1 month postloss to 8.6% at 24 months postloss (data not shown).

We previously [31] used latent-class growth analysis (LCGA) to identify 7 distinct gender- and age-adjusted depressive-symptom trajectories by their timing, intensity, and duration of depressive symptoms (prevalence): minimal-impact resilience (20.4%), recovery (34.0%), preloss-depressive only (21.6%), delayed symptomatic (9.1%), relief (5.9%), prolonged symptomatic (6.5%), and chronically persistent distressed (2.5%) (Supplemental Fig. 1, Appendix 2).

#### Independent variables

Drawing from the stress-appraisal-coping model, modifiable factors associated with caregivers’ membership in the seven depressive-symptom trajectories were examined in three domains [25]: (1) stressors, (2) stress appraisal, and (3) available resources*.* Caregiver responses to variables in these categories could differ at various data-collection times, making them time-varying variables.

Stressors indicated as objective caregiving demands were measured by the amount of assistance provided in personal care, homemaking, transportation, and health care on a 4-point scale (1=none at all to 4=a lot) [33]. Caregiving demand scores range=4-16; higher scores indicate greater caregiving demands. To comprehensively reflect the dynamic nature of objective caregiving demands throughout the patient’s last 6 months, objective-caregiving-demand trajectories were identified using LCGA with a continuous latent-class indicator (total objective caregiving demand score) to identify stressor-trajectories experienced by caregivers over their entire EOL caregiving period. Four objective-caregiving-demand trajectories were identified by their intensity and patterns of change: profound, decreasing, increasing, and low objective caregiving demands using the LCGA procedures and criteria for selecting the optimal number of classes prior indicated [31].

Stress appraisal refers to appraisal of caregiving impact. The 24-item Caregiver Reaction Assessment (CRA) [34] measures EOL-caregiving impact on caregivers’ schedule, health, and finances, as well as caregiver esteem in providing care (rewarding or causing resentment), and lack of family support. Total scores range from 24 to 120. Higher scores represent stronger negative appraisal of EOL-caregiving impact. Similarly, to reflect the dynamic nature of subjective appraisal of caregiving impact, we identified caregivers’ CRA trajectories using LCGA with a continuous latent-class indicator (total CRA score) to identify stress-appraisal trajectories experienced by caregivers over their entire EOL caregiving period. Four CRA trajectories were identified and were named low, moderate, high, and substantial caregiving burden using the LCGA procedures and criteria for selecting the optimal number of classes previously indicated [31]. These four trajectories differed in their CRA-score levels. Detailed information about use of latent class-growth analysis to identify optimal number of distinct objective-caregiving-demand and CRA trajectories is available upon request via the corresponding author.

Caregivers’ available resources were internal coping capacity and external social support. Caregivers’ coping capacity was measured by the 13-item sense of coherence scale [35]. Sense of coherence, an important coping capacity for adjusting to stressors to restore homeostasis, has three components: comprehensibility (belief that life is structured and predictable), meaningfulness (life is challenging but worthwhile), and manageability (resources suffice to manage challenges) [35]. Total sense of coherence scale scores range from 13 to 91; higher scores indicate stronger sense of coherence.

Caregivers’ perceived social support was measured by the 19-item Medical Outcomes Study Social Support Survey (MOS-SSS) [36], which assesses emotional, informational, tangible, and affectionate support, as well as positive social interaction. Total scores are computed on a 0-100 scale; higher scores indicate stronger perceived social support.

#### Covariates

Contextual factors [25] were treated as covariates and included caregivers’ marital status (married vs unmarried), relationship with the patient (spouse/adult child/other*)*, educational attainment (< vs > senior high school), financial status (making ends meet [yes/no])*,* comorbidity (yes/no), and awareness of the patient’s prognosis. Caregiver prognostic awareness was measured at each assessment by asking caregivers whether they knew their relative’s prognosis, and if so, whether the disease (1) was curable; (2) might recur in the future, but the patient’s life was not currently in danger; or (3) could not be cured and the patient would probably die soon. Caregivers were recognized as accurately knowing the patient’s prognosis only if they chose option 3; inaccurate prognostic awareness reflected not knowing the patient’s prognosis or choosing option 1 or 2.

### Statistical analysis

Potential factors associated with caregivers’ membership in the seven distinct depressive-symptom trajectories (see Independent variables) were examined by multinomial logistic regressions while controlling for covariates/contextual factors. Multinomial logistic regressions are flexible enough to allow the researcher to alter the reference category, allowing for all possible group comparisons, while the overall model statistics remain unchanged. Preloss scores of internal and external resources were measured at the first assessment within patient’s last 6 months, whereas postloss scores were measured 1-month postloss. This timeline reflected the two-phase piecewise estimation used to model changes in depressive symptoms from EOL caregiving through the first 2 bereavement years, with time of transitioning into EOL caregiving and time of loss serving as major life transitions for caregivers [31]. The regression estimate for each independent variable in the multinomial logistic-regression models was exponentiated to transform to adjusted odds ratio (AOR) with 95% confidence interval (CI).

## Results

### Factors associated with distinct depressive-symptom trajectories

Details of associations between variables and membership in each depressive-symptom trajectory are in Tables 1, 2, 3 and 4, specifically relative to the minimal-impact-resilience (Table 1), recovery (Table 2), preloss-depressive-only (Table 3), delayed-symptomatic (Table 4), relief (Table 4), and prolonged-symptomatic (Table 3) trajectories, respectively. Descriptive data of each potential factor associated with the seven distinct depressive-symptom trajectories are Table 6 in Appendix 3.

**Table 1:** Factors associated with distinct depressive-symptom trajectories from end-of-life caregiving through the first 2 years of bereavement--Minimal-impact resilience vs others

Potential Factor	Relative odds of belonging to the indicated trajectory category compared to the minimal-impact resilience trajectory																							
	Recovery				Preloss depressive only				Delayed symptomatic				Relief				Prolonged symptomatic				Persistently distressed			
	AOR	95% CI		p	AOR	95% CI		p	AOR	95% CI		p	AOR	95% CI		p	AOR	95% CI		p	AOR	95% CI		p
**Stressors**																								
**Objective-caregiving-demand trajectory**																								
Profound	**6.105**	**2.451**	**15.203**	**<.001**	**15.665**	**5.432**	**45.174**	**<.001**	**10.123**	**2.751**	**37.250**	**<.001**	**30.331**	**4.389**	**209.594**	**.001**	**22.701**	**4.058**	**127.001**	**<.001**	**25.561**	**1.648**	**396.467**	**.020**
Decreasing	0.747	0.210	2.648	.651	1.894	0.488	7.343	.356	**---**	**---**	**---**	**---**	5.079	0.497	51.873	.170	1.079	0.104	11.182	.949	4.411	.159	122.447	.381
Increasing	1.762	0.890	3.485	.104	**2.879**	**1.212**	**6.840**	**.017**	1.470	0.492	4.389	.490	2.876	0.437	18.945	.272	1.545	0.316	7.552	.591	1.923	.146	25.363	.619
Low	Ref				Ref				Ref				Ref				Ref				Ref			
**Stress appraisal**																								
**Subjective-caregiving-burden trajectory**																								
Low	2.110	0.149	29.786	.581	0.255	0.020	3.336	.298	1.605	0.068	38.013	.770	0.089	0.003	2.429	.152	0.479	0.013	17.087	.686	**---**	**---**	**---**	**---**
Moderate	2.090	0.165	26.507	.569	0.445	0.041	4.802	.505	1.656	0.094	29.320	.731	0.112	0.009	1.445	.093	0.735	0.050	10.890	.823	0.850	0.039	18.724	.918
High	1.836	0.140	24.158	.644	1.305	0.119	14.350	.828	1.110	0.061	20.224	.944	0.223	0.018	2.814	.246	1.005	0.072	14.040	.997	1.118	0.065	19.135	.939
Substantial	Ref				Ref				Ref				Ref				Ref				Ref			
**Psychosocial Resources**																								
**SOC**																								
Preloss	1.013	0.989	1.038	.291	**0.942**	**0.919**	**0.966**	**<.001**	0.986	0.953	1.019	.396	**0.901**	**0.867**	**0.936**	**<.001**	**0.963**	**0.929**	**0.999**	**.042**	0.962	0.911	1.016	.167
Postloss	**0.933**	**0.912**	**0.953**	**<.001**	**0.968**	**0.946**	**0.991**	**.007**	**0.933**	**0.906**	**0.960**	**<.001**	**0.954**	**0.924**	**0.985**	**.004**	**0.908**	**0.879**	**0.938**	**<.001**	**0.871**	**0.824**	**0.921**	**<.001**
**Social support**																								
Preloss	1.015	0.988	1.043	.265	1.023	.993	1.053	.132	1.029	0.989	1.070	.160	1.034	0.990	1.081	.134	1.031	0.987	1.077	.174	0.996	0.927	1.071	.923
Postloss	**0.921**	**0.894**	**0.949**	**<.001**	**0.944**	**0.914**	**0.976**	**.001**	**0.883**	**0.849**	**0.920**	**<.001**	**0.954**	**0.910**	**0.999**	**.047**	**0.859**	**0.820**	**0.899**	**<.001**	**0.883**	**0.828**	**0.943**	**<.001**

Subjective caregiving burden was measured by the Caregiver Reaction Assessment

Social support was measured by the Medical Outcomes Study Social Support Survey

Bolds indicate statistically significant differences. “---“ indicates no AOR or 95% CI could be estimated due to insufficient small sample

*Abbreviations: SOC* Sense of coherence, *AOR* adjusted odds ratios, *CI* confidence interval, Ref references

**Table 2:** Factors associated with distinct depressive-symptom trajectories from end-of-life caregiving through the first 2 years of bereavement- Recovery vs others

Potential Factor	Relative odds of belonging to the indicated trajectory category compared to the recovery trajectory																			
	Preloss depressive only				Delayed symptomatic				Relief				Prolonged symptomatic				Persistently distressed			
	AOR	95% CI		p	AOR	95% CI	p		AOR	95% CI		p	AOR	95% CI		p	AOR	95% CI		p
**Stressors**																				
** Objective-caregiving-demand trajectory**																				
Profound	**2.566**	**1.058**	**6.223**	**.037**	1.658	0.539	5.097	.377	4.968	0.797	30.977	.086	3.718	0.775	17.830	.101	4.187	.299	58.582	.287
Decreasing	2.537	0.674	9.544	.168	**---**	**---**	**---**	**---**	6.804	0.689	67.186	.101	1.445	0.151	13.806	.749	5.909	.227	153.564	.285
Increasing	1.634	0.719	3.714	.241	0.834	0.301	2.311	.728	1.633	0.256	10.405	.604	0.877	0.193	3.993	.865	1.092	.087	13.687	.946
Low	Ref				Ref				Ref				Ref				Ref			
**Stress appraisal**																				
** Subjective-caregiving-burden trajectory**																				
Low	**0.121**	**0.021**	**0.681**	**.017**	0.761	0.074	7.848	.818	**0.042**	**0.003**	**0.649**	**.023**	0.227	0.013	3.902	.307	**---**	**---**	**---**	**---**
Moderate	**0.213**	**0.050**	**0.910**	**.037**	0.792	0.110	5.705	.817	**0.053**	**0.009**	**0.313**	**.001**	0.351	0.065	1.909	.226	0.407	0.041	4.017	.441
High	0.711	0.172	2.944	.638	0.605	0.082	4.439	.621	**0.121**	**0.023**	**0.630**	**.012**	0.547	0.113	2.656	.455	0.609	0.090	4.138	.612
Substantial	Ref				Ref				Ref				Ref				Ref			
**Psychosocial Resources**																				
** SOC**																				
Preloss	**0.930**	**0.911**	**0.949**	**<.001**	0.973	0.946	1.001	.058	**0.889**	**0.859**	**0.922**	**<.001**	**0.951**	**0.922**	**0.981**	**.001**	**0.950**	**0.903**	**1.000**	**.049**
Postloss	**1.038**	**1.021**	**1.057**	**<.001**	1.000	0.978	1.023	.970	1.023	0.995	1.052	.102	**0.974**	**0.949**	**1.000**	**.049**	**0.934**	**0.887**	**0.984**	**.011**
** Social support**																				
Preloss	1.007	0.983	1.032	.553	1.013	0.979	1.048	.457	1.019	.978	1.061	.373	1.015	.977	1.055	.440	0.981	0.916	1.051	.589
Postloss	1.025	1.000	1.051	.051	**0.959**	**0.929**	**0.990**	**.010**	1.035	.994	1.078	.097	**0.932**	**0.897**	**0.969**	**<.001**	0.959	0.903	1.018	.167

Subjective caregiving burden was measured by the Caregiver Reaction Assessment

Social support was measured by the Medical Outcomes Study Social Support Survey

Bolds indicate statistically significant differences. “---“ indicates no AOR or 95% CI could be estimated due to insufficient small sample

*Abbreviations: SOC* Sense of coherence, *AOR* adjusted odds ratios, *CI* confidence interval, *Ref* references

**Table 3:** Factors associated with distinct depressive-symptom trajectories from end-of-life caregiving through the first 2 years of bereavement- Preloss-depressive only vs others

Potential Factor	Relative odds of belonging to the indicated trajectory category compared to the preloss-depressive only trajectory																Relative odds of belonging to the persistently distressed compared to the prolonged symptomatic trajectory			
	Delayed symptomatic				Relief				Prolonged symptomatic				Persistently distressed				Persistently distressed			
	AOR	95% CI	p		AOR	95% CI		p	AOR	95% CI		p	AOR	95% CI		p	AOR	95% CI		p
**Stressors**																				
** Objective-caregiving-demand trajectory**																				
Profound	0.646	0.182	2.289	.499	1.936	0.309	12.130	.480	1.449	0.276	7.612	.661	1.632	0.110	24.168	.722	1.126	0.062	20.402	.936
Decreasing	**---**	**---**	**---**	**---**	2.682	0.294	24.432	.381	0.570	0.060	5.441	.625	2.329	0.090	60.288	.610	4.088	0.118	142.025	.437
Increasing	0.511	0.159	1.643	.260	0.999	0.153	6.511	.999	0.537	0.107	2.691	.449	0.668	0.050	8.900	.760	1.245	0.075	20.652	.878
Low	Ref				Ref				Ref				Ref				Ref			
**Stress appraisal**																				
** Subjective-caregiving-burden trajectory**																				
Low	6.288	0.565	70.002	.135	0.350	0.026	4.755	.431	1.877	0.104	333.748	.669	**---**	**---**	**---**	**---**	**---**	**---**	**---**	**---**
Moderate	3.720	0.536	25.821	.184	0.251	0.055	1.150	.075	1.650	0.326	8.360	.545	1.909	0.209	17.450	.567	1.157	0.122	10.949	.899
High	0.851	0.127	5.714	.868	**0.171**	**0.045**	**0.648**	**.009**	0.770	0.186	3.183	.718	0.857	0.148	4.972	.863	1.113	0.199	6.218	.903
Substantial	Ref				Ref				Ref				Ref				Ref			
**Psychosocial Resources**																				
** SOC**																				
Preloss	**1.046**	**1.016**	**1.078**	**.003**	**0.957**	**0.925**	**0.989**	**.010**	1.023	0.990	1.056	.174	1.022	0.970	1.076	.418	0.999	0.947	1.054	.976
Postloss	**0.963**	**0.940**	**0.988**	**.004**	0.986	0.959	1.012	.289	**0.938**	**0.912**	**0.964**	**<.001**	**0.900**	**0.853**	**0.949**	**<.001**	0.959	0.908	1.014	.139
** Social support**																				
Preloss	1.006	0.970	1.043	.762	1.011	0.972	1.052	.577	1.008	0.969	1.049	.697	0.974	0.909	1.044	.459	0.967	0.899	1.039	.354
Postloss	**0.935**	**0.903**	**0.969**	**<.001**	1.010	0.971	1.051	.630	**0.909**	**0.873**	**0.947**	**<.001**	**0.935**	**0.881**	**0.993**	**.030**	1.029	0.966	1.095	.375

Subjective caregiving burden was measured by the Caregiver Reaction Assessment;

Bolds indicate statistically significant differences. “---“ indicates no AOR or 95% CI could be estimated due to insufficient small sample

Social support was measured by the Medical Outcomes Study Social Support Survey

*Abbreviations: SOC* Sense of coherence, *AOR* adjusted odds ratios, *CI* confidence interval, *Ref* references

**Table 4:** Factors associated with distinct depressive-symptom trajectories from end-of-life caregiving through the first 2 years of bereavement- Delayed symptomatic and relief vs others

Potential Factor	Relative odds of belonging to the indicated trajectory category compared to the delayed symptomatic trajectory												Relative odds of belonging to the indicated trajectory category compared to the relief trajectory							
	Relief				Prolonged symptomatic				Persistently distressed				Prolonged symptomatic				Persistently distressed			
	AOR	95% CI		p	AOR	95% CI		p	AOR	95% CI		p	AOR	95% CI		p	AOR	95% CI		p
**Stressors**																				
** Objective-caregiving-demand trajectory**																				
Profound	2.996	.391	22.963	.291	2.242	0.386	13.031	.368	2.525	0.159	40.071	.511	0.748	0.076	7.395	.804	0.843	0.037	19.055	.914
Decreasing	**---**	**---**	**---**	**---**	**---**	**---**	**---**	**---**	**---**	**---**	**---**	**---**	0.212	0.012	3.759	.291	0.868	0.022	34.664	.940
Increasing	1.957	.258	14.848	.516	1.051	0.195	5.672	.954	1.309	0.094	18.262	.841	0.537	0.054	5.353	.596	0.669	0.031	14.285	.797
Low	Ref				Ref				Ref				Ref				Ref			
**Stress appraisal**																				
** Subjective-caregiving-burden trajectory**																				
Low	0.056	0.002	1.370	.077	0.298	0.012	7.391	.460	**---**	**---**	**---**	**---**	5.356	.153	187.751	.355	---	---	---	---
Moderate	**0.067**	**0.008**	**0.598**	**.015**	0.444	0.058	3.394	.434	0.513	0.039	6.745	.612	**6.574**	**1.005**	**43.013**	**.049**	7.608	0.685	84.542	.099
High	0.201	0.025	1.594	.129	0.905	0.129	6.329	.920	1.007	0.106	9.569	.995	4.510	.907	22.424	.066	5.017	0.739	34.051	.099
Substantial	Ref				Ref				Ref				Ref				Ref			
**Psychosocial Resources**																				
** SOC**																				
Preloss	**0.914**	**0.877**	**0.953**	**<.001**	0.977	0.942	1.014	.215	0.976	0.924	1.032	.393	**1.069**	**1.025**	**1.114**	**.002**	**1.068**	**1.007**	**1.132**	**.027**
Postloss	1.023	0.990	1.057	.177	0.974	0.944	1.004	.092	**0.934**	**0.884**	**0.987**	**.015**	**0.952**	**0.920**	**0.985**	**.005**	**0.913**	**0.863**	**0.966**	**.002**
** Social support**																				
Preloss	1.006	.958	1.056	.821	1.002	0.957	1.049	.922	0.969	0.900	1.042	.396	0.997	0.948	1.048	.896	0.963	0.893	1.039	.331
Postloss	**1.079**	**1.029**	**1.132**	**.002**	0.972	0.930	1.016	.209	1.000	0.938	1.066	.997	**0.900**	**0.857**	**0.946**	**<.001**	**0.926**	**0.867**	**0.990**	**.024**

Subjective caregiving burden was measured by the Caregiver Reaction Assessment

Social support was measured by the Medical Outcomes Study Social Support Survey

Bolds indicate statistically significant differences. “---“ indicates no AOR or 95% CI could be estimated due to insufficient small sample

*Abbreviations: SOC* Sense of coherence, *AOR* adjusted odds ratios, *CI* confidence interval, *Ref* references

In the stressor domain, relative to caregivers with low objective caregiving demands, those with profound objective caregiving demands were more likely to be in the more distressing depressive-symptom trajectories than in the minimal-impact-resilience trajectory (Table 1) and in the preloss-depressive-only than in the recovery trajectory (Table 2). Furthermore, caregivers with increasing objective caregiving demands were more likely than those with low objective caregiving demands to be in the preloss-depressive-only than in the minimal-impact-resilience trajectory (Table 1).

In the stress-appraisal domain, compared to those with substantial caregiving burden, caregivers with low and moderate caregiving burden were less likely to be in the preloss-depressive-only and relief trajectories than in the recovery trajectory (Table 2). Caregivers in the moderate CRA-trajectory were less likely than those with substantial caregiving burden to be in the relief trajectory than in the delayed-symptomatic and prolonged-symptomatic trajectories (Table 4), whereas those in the high CRA-trajectory were less likely than those with substantial caregiving burden to be in the relief trajectory than in the recovery and preloss-depressive-only trajectories (Tables 2 and 3).

In the psychosocial-resource domain, higher reported preloss coping capacity (sense-of-coherence score) increased the odds that caregivers would be in the recovery trajectory than in all other trajectories, except the delayed-symptomatic trajectory (Table 2). Caregivers with higher preloss sense-of-coherence scores were also less likely to be in the preloss-depressive-only, relief, and prolonged-symptomatic trajectories than in the minimal-impact-resilience trajectory (Table 1) as well as less likely to be in the relief trajectory than in the preloss-depressive-only (Table 3), delayed-symptomatic, prolonged-symptomatic, and chronically persistent-distressed (Table 4) trajectories but were more likely to be in the delayed-symptomatic than in the preloss-depressive-only trajectory (Table 3). Moreover, stronger postloss sense of coherence was linked more to the minimal-impact-resilience trajectory than to all others (Table 1). Furthermore, these caregivers were less likely to be in the prolonged-symptomatic and chronically persistent-distressed trajectories than in the recovery (Table 2), preloss-depressive-only (Table 3), delayed-symptomatic (Table 4), and relief trajectories (Table 4) but more likely to be in the preloss-depressive-only trajectory than in the recovery (Table 2) and delayed-symptomatic (Table 3) trajectories.

Perceived preloss social support was not associated with caregivers’ membership in depressive-symptom trajectories, but stronger perceived postloss social support increased the odds that caregivers would be in the minimal-impact-resilience trajectory relative to all other trajectories (Table 1). Furthermore, these caregivers were less likely to be in the delayed trajectory than in the recovery (Table 2), preloss-depressive-only (Table 3) and relief (Table 4) trajectories, in the prolonged-symptomatic than in the recovery trajectory (Table 2), and in the prolonged-symptomatic and chronically persistent-distressed trajectories than in the preloss-depressive-only (Table 3) and relief (Table 4) trajectories.

## Discussion

Our study examined modifiable factors associated with caregivers’ membership in the distinct depressive-symptom trajectories from EOL caregiving through the first 2 bereavement years based on the stress-appraisal-coping model [25]. All proposed variables, except preloss perceived social support, were associated with caregivers’ membership in depressive-symptom trajectories. The nature of the relationship between preloss social support and depressive-symptom trajectories is inconsistent in the literature with reports of no association, [19] association with group membership during bereavement [20], and consistent association with depressive-symptom trajectories during caregiving [13, 14, 15]. Whether the association of preloss social support with caregivers’ membership in depressive-symptom trajectories from EOL caregiving through the first 2 bereavement years was mediated by other variables warrants further research.

Our study’s use of data from before and after patient death confirmed both the cumulative-stress (wear-and-tear model) and stress-reduction (relief model) perspectives of caregiving effects on bereavement adjustment [28]. Profound objective caregiving demands consistently and cumulatively burden caregivers and do not end with the patient’s death but exhaust caregivers, leaving them vulnerable to the highly stressful death of their relative, which would explain the stronger link to more distressing depressive-symptom trajectories than to the minimal-impact-resilience trajectory (Table 1). Similarly, a wear-and-tear effect has been reported for caregiving role overload on postloss depressive-symptom trajectories [18, 20].

In contrast, caregivers with substantial caregiving burden were more likely than those with moderate caregiving burden to be in the relief trajectory than in delayed-symptomatic and prolonged symptomatic trajectories (Table 4). Furthermore, they were more likely than those with lighter caregiving burden to be in the preloss-depressive-only or relief trajectories (Table 2), in which moderate-profound depressive symptoms either resolved completely or improved dramatically after the patient’s death, respectively, than in the recovery trajectory. Our results support the relief model of bereavement [29], which suggests that after the difficulties of EOL caregiving, patient death brings the caregiver relief and thus better bereavement outcomes [29]. In a Confucian context, death of one’s relative after substantial caregiving burden may bring a sense of fulfillment of filial duty and relief not only from their heavy caregiving burden but also from the end of their relative’s suffering, thereby explaining fewer depressive symptoms.

Personal coping capacity/adaptive coping strategies have consistently emerged as robust predictors to buffer long-term negative outcomes following aversive circumstances [18, 20, 26]. We measured sense of coherence when caregivers first transitioned into EOL caregiving and bereavement to reflect the major life transitions for caregivers; here we confirm that both measures were associated with caregivers’ membership in distinct depressive-symptom trajectories. We found that caregivers with stronger preloss sense of coherence were less likely to be in (1) trajectories characterized by moderate-to-severe preloss depressive symptoms than in the recovery trajectory (Table 2), (2) preloss-depressive-only, relief, and prolonged-symptomatic trajectories than in the minimal-impact-resilience trajectory (Table 1), and (3) the relief than preloss-depressive-only and delayed-symptomatic trajectories (Tables 3-4), both with substantially fewer preloss depressive symptoms. Furthermore, caregivers with stronger postloss sense of coherence were more likely to be in the minimal-impact-resilience trajectory (Table 1) than to all others as well as to the recovery, preloss-depressive-only, and relief (Tables 2, 3 and 4) trajectories than to trajectories characterized by moderate-to-profound postloss depressive symptoms without complete recovery over the first 2 bereavement years, (i.e., prolonged-symptomatic and persistently-distressed trajectories). Caregivers with higher pre- and postloss sense of coherence were at lower risk for stress from EOL caregiving and were more likely to be in the resilient or milder-and-transient depressive-symptom trajectories, which confirms that personal coping capacity matters when individuals face adversity and life challenges [35]. However, we could not explain why stronger preloss sense of coherence increased odds for the delayed-symptomatic, prolonged-symptomatic, or chronically persistent-distressed trajectories relative to the preloss-depressive-only (Table 3) and relief (Table 4) trajectories, respectively, warranting validation. However, caregivers with high sense of coherence levels (i.e., extremely positive schemas about themselves and the world) may be at higher risk for developing more distressing depressive-symptom trajectories, because adverse, highly stressful caregiving events cannot easily be integrated into their existing schemas [38]. This assertion warrants further in-depth investigations, preferably by qualitative research.

A similar pattern to the role of sense of coherence in easing depressive symptoms during bereavement was observed for perceived postloss social support, as reported [17, 18, 27]. We found that caregivers with stronger perceived postloss social support were more likely to be in the minimal-impact-resilience trajectory relative to all other trajectories (Table 1). Furthermore, caregivers who perceived stronger postloss social support were less likely to be in long-lasting distressing depressive-symptom trajectories than in the recovery, preloss-depressive-only, and relief trajectories (Tables 2, 3 and 4), as well as more likely to be in the preloss-depressive-only or relief trajectories characterized by moderate-to-profound preloss depressive symptoms that subsided quickly before patient death or 6 months postloss than in the delayed trajectories (Tables 3 and 4). Stronger perceived social support reflects the connectedness, emotional comfort, and practical/instrumental resources bereaved caregivers might find in their social network to buffer their grief at losing a longstanding relationship with the deceased and to help them cope with challenges inherent in living without the deceased [26, 27], thereby easing their depressive symptoms.

### Study Strengths and Limitations

This study’s strengths lie in its investigation of modifiable factors associated with caregivers to distinct depressive-symptom trajectories from EOL caregiving through the first 2 years of bereavement using information assessed before and after patient death. These strengths allowed us to disentangle the cumulative-stress (wear-and-tear model) and stress-reduction (relief model) perspectives [28] of EOL-caregiving effects on bereavement and to identify protective and vulnerability factors modifiable by high-quality EOL care. Despite our study’s theoretical contributions and methodological advantages, several limitations warrant mention. Caregivers were sampled by convenience from a single Taiwanese hospital, possibly limiting generalization of our findings to national and international target populations, especially considering cultural variations in grief reactions towards losing a relative [39]. Our results cannot be generalized to bereaved caregivers who lose their relative due to other diseases or sudden/traumatic death. We measured depressive symptoms with the CES-D, probably overestimating the severity of depressive symptoms but avoiding overlooking caregivers’ need for psychological support or treatment. Depressive-symptom trajectories were explored only through the first 2 years of bereavement. We explored main effects of each identified variable based on the stress-appraisal-coping model [25] on caregivers’ membership in depressive-symptom trajectories. However, we did not find major associations with perceived social support when caregivers first transitioned into caregiving [20], different patterns of objective caregiving demands [18, 20], or different levels of subjective caregiving burden [40], as commonly reported. We speculate that the roles played by these variables in associations with depressive-symptom trajectories were mediated by sense of coherence [41] and perceived postloss social support [37], which has not yet been explored. Despite our large sample, some categories of our outcome and independent variables may not have had sufficient subjects to appropriately estimate associations in our multinomial logistic regression models. We can never infer a causal-effect relationship nor exclude the possible impact of unmeasured variables, e.g., pre-caregiving emotional status, receipt of mental health services pre- and postloss, or quality of caregiver-patient relationship, commonly found in observational studies.

## Conclusions and clinical implications

Family caregivers of Taiwanese advanced cancer patients follow distinct depressive-symptom trajectories from EOL caregiving through bereavement. Clinical interventions should be prioritized to interrupt the three unfavorable long-lasting and delayed high-level depressive-symptom trajectories, to prevent burnout during caregiving for caregivers in the preloss-depressive-only trajectory, and to facilitate rapid return to healthy emotional functioning for bereaved in the relief trajectory. Furthermore, our findings showed that these trajectories are linked to preloss caregiving demands, appraisal of EOL-caregiving impact, internal coping capacity, and perceived social support. Profound caregiving demands may cumulatively exhaust caregivers, whereas caregivers who appraise EOL caregiving as a substantial burden may feel relief and experience fewer depressive symptoms once their relative dies. Stronger internal coping capacity and external social support may buffer the tremendous stress from EOL caregiving and the permanent loss of a relative. Healthcare professionals should be sensitive to the caregiving needs of caregivers with heavy caregiving demands and provide effective interventions. For example, caregivers’ coping capacity could be boosted and their social support enhanced to help them adequately manage daily caregiving to relieve the emotional toll before patient death and throughout bereavement. These actionable opportunities for high-quality EOL care can facilitate caregivers’ adjustment to the stress of EOL caregiving and bereavement, thus benefiting caregivers and society.

### Supplementary Information

**Additional file 1.**

## Data Availability

Sharing anonymized data from this study is restricted due to ethical and legal constraints. Data contains sensitive personal health information, which is protected under The Personal Data Protection Act in Taiwan, thus making all data requests subject to Institutional Review Board (IRB) approval. Per the IRB of the study hospital, the data that support the findings of this study are restricted for transmission to those outside the primary investigative team. Data sharing with investigators outside the team requires IRB approval. All requests for anonymized data will be reviewed by the research team and then submitted to the Chang Gung Medical Foundation IRB for approval. Detailed information about data used in this study is available upon request via the corresponding author.

## Appendix 1

Table 5

**Table 5:** Participants’ characteristics (*N* = 661)

Characteristic		***n***^***a***^	%^**b**^	Characteristic	***n***^***a***^	%^**b**^	
Gender				Marital status (*n* = 661)			
Male		191	28.9	Unmarried	111	16.8	
Female		470	71.1	Married	550	83.2	
Age (years) (*n* = 655)				Financial sufficiency (*n* = 639)			
Mean (SD)		52.14 (12.67)		Making ends meet	530	82.9	
Relationship to patient (*n* = 660)				Financial strain	109	17.1	
Spouse		431	65.2	Accurate prognostic awareness			
Child		134	20.3	No	50	7.6	
Other		96	14.5	Yes, time proximity to death, days			
Educational attainment (*n* = 654)				1-30	165	25.0	
< Senior high school		519	79.4	31-60	113	17.1	
> Senior high school		135	20.6	61-90	83	12.6	
Chronic disease (*n* = 661)				91-120	56	8.5	
Yes		221	33.4	121-150	49	7.4	
No		440	66.6	151-180	145	21.9	
OCD trajectories				CRA trajectories			
Profound		220	33.3	1	102	15.4	
Decreasing		63	9.5	2	377	57.0	
Increasing		274	41.4	3	135	20.5	
Low		104	15.7	4	47	7.1	
Preloss initial assessment				Final assessment before bereavement			
Variable	# assessments	Mean	SD	Variable	# assessments	Mean	SD
SOC	660	60.65	17.10	SOC	654	57.13	18.29
MOS-SSS	660	63.06	13.57	MOS-SSS	661	61.48	13.23

*OCD* Objective caregiving demands, *CRA* Caregiver Reaction Assessment, *SOC* Sense of coherence, *MOS-SSS* Medical Outcomes Study Social Support Survey

^a^The total number of cases for each characteristic may not equal the sample size due to missing data

^b^Due to the rounding error, the total percentage of each characteristic may not equal to 100%

## Appendix 2

### Description of the identified depressive-symptom trajectories

The minimal-impact-resilience trajectory showed a stable, low depressive-symptom level from caregiving through the first 2 bereavement years, with mild and transient perturbations around the patient’s death. For the recovery trajectory, CES-D exceeded the threshold 1-month preloss, peaked in the first month postloss, then dropped below threshold around 6-7 months postloss. The preloss-depressive-only trajectory was characterized by slight-to-moderate depressive symptoms during EOL caregiving, subsiding quickly to near threshold 1-month postloss, thereafter decreasing gradually. Caregivers in the delayed symptomatic trajectory initially had slight-to-moderate depressive symptoms that gradually intensified to moderate-to-high levels around the patient’s death, thereafter trending slowly downward but increasing slightly since 18 months postloss without a complete resolution over the first 2 bereavement years. Relief-group caregivers increasingly suffered moderate-to-profound depressive symptoms while caregiving, but as the patient’s death approached, their depressive symptoms started subsiding significantly and dropped below threshold around 6-7 months postloss. The prolonged-symptomatic trajectory was characterized by preloss moderate-to-severe depressive-symptoms, peaking 1-month postloss, thereafter declining steadily and resolving completely at the end of the first 2 bereavement years. Caregivers in the chronically persistent-distressed trajectory suffered profound depressive symptoms during EOL caregiving, their depressive-symptom level peaked 1-month postloss, then improved over the first 2 bereavement years but remained well above threshold.

Of note, only caregivers’ depressive symptoms were measured as the manifestation of grief reactions toward the forthcoming death or the loss of their relative without measuring grief symptoms more generally. Furthermore, the recovery trajectory is named to reflect the trajectory that returns to the normal level of depressive symptoms within 6 months postloss and has no implication that an individual can “recover” from a significant interpersonal loss.

* Note: from Wen FH, Chou WC, Hou MM, et al. Depressive-symptom trajectories from end-of-life caregiving through the first 2 years of bereavement for family caregivers of advanced cancer patients. J Pain Symptom Manage.2021; 62:699-708.

## Appendix 3

Table 6

**Table 6:** Modifiable factors of caregivers’ distinct depressive-symptom trajectories

Depressive-symptom trajectory Factors	Minimal-impact resilience	Recovery	Preloss depressive only	Delayed symptomatic	Relief	Prolonged symptomatic	Persistently distressed
**Stressor: Objective-caregiving-demand trajectory (%)**							
Low	29.5	15.6	10.1	14.3	5.9	0.0	7.1
Increasing	52.1	50.3	49.7	41.4	23.5	35.0	28.6
Decreasing	6.8	1.5	8.7	4.3	9.8	0.0	14.3
Profound	11.6	32.7	31.5	40.0	60.8	65.0	50.0
**Stress appraisal: Subjective-caregiving-burden trajectory (%)**							
Low	32.2	16.6	8.7	7.1	3.9	0.0	0.0
Moderate	60.3	69.3	51.0	64.3	27.5	25.0	28.6
High	6.8	12.1	35.6	22.9	33.3	35.0	42.9
Substantial	0.7	2.0	4.7	5.7	35.3	40.0	28.6
**Psychosocial Resources**							
**Sense of coherence (M [SD])**							
Preloss	70.7(13.5)	65.4(13.9)	55.8(15.2)	59.3(14.4)	39.4(15.8)	44.9(19.0)	45.1(16.7)
Postloss	68.7(13.4)	56.0(17.9)	58.3(16.4)	52.1(16.5)	47.6(17.9)	34.1(16.3)	34.6(15.3)
**Perceived social support (M [SD])**							
Preloss	68.7(12.6)	64.4(12.0)	61.6(13.6)	60.5(11.4)	55.7(16.5)	52.3(15.0)	54.7(12.6)
Postloss	68.7(11.6)	60.2(11.6)	62.9(10.8)	53.9(12.8)	60.9(15.6)	48.5(17.1)	49.4(17.4)

Subjective caregiving burden was measured by the Caregiver Reaction Assessment; Sense of coherence was measured by the SOC-13 scale

Social support was measured by the Medical Outcomes Study Social Support Survey
