# Supplementary material for: Modifiable factors of depressive-symptom trajectories from caregiving through bereavement
Source: BMC Palliat Care. 2022 Sep 7;21:156. doi: 10.1186/s12904-022-01045-9 (PMC9454199; doi:10.1186/s12904-022-01045-9)
Supplement: Supplementary file 1 — Additional file 1. [file 12904_2022_1045_MOESM1_ESM.docx]

**Supplemental** **Figure 1**. Depressive-symptom trajectories from end-of-life caregiving through the first 2 bereavement years

CESD Scores

Trajectory 1 (C1): Minimal impact resilience; Trajectory 2 (C2): Recovery; Trajectory 3: Preloss-depressive only (C3); Trajectory 4 (C4): Delayed symptomatic; Trajectory 5 (C5): Relief; Trajectory 6 (C6): Prolonged symptomatic; Trajectory 7 (C7): Chronically persistent distressed.

Note: The horizontal black line over CES-D score at 16 indicates the threshold for severe depressive symptoms.
